# Shoulder joint replacement can improve quality of life and outcome in patients with dysmelia: a case series

**DOI:** 10.1186/s12891-016-1031-x

**Published:** 2016-04-26

**Authors:** Tobias Peter Merkle, Nicholas Beckmann, Tom Bruckner, Felix Zeifang

**Affiliations:** Klinikum Stuttgart, Department of Orthopedics and Trauma Surgery, Katharinenhospital Stuttgart, Academic Teaching Hospital of University Tübingen, Kriegsbergstraße 60, 70174 Stuttgart, Germany; Department of Orthopedics, Trauma Surgery and Paraplegiology, Heidelberg University Hospital, Schlierbacher Landstraße 200a, 69118 Heidelberg, Germany; Medical Biometry and Informatics, Heidelberg University, INF 305, 69120 Heidelberg, Germany

**Keywords:** Thalidomide, Phocomelia, Glenohumeral dysmelia, Osteoarthritis, Arthroplasty, Stemless shoulder prosthesis

## Abstract

**Background:**

Arthroplasty is a proven treatment option for glenohumeral osteoarthritis. Common indications include primary or posttraumatic osteoarthritis, avascular necrosis of the humeral head, rotator cuff tear arthropathy and rheumatoid osteoarthritis. Arthroplasty is rarely performed among patients with glenohumeral dysmelia. An overuse of the upper limb in patients with thalidomide-induced phocomelia and people with similar congenital deformities like dysmelia results in premature wear of the shoulder joint. This study aims to evaluate our experience with cases of glenohumeral osteoarthritis caused by dysmelia and treated with arthroplasty. To date, few reports on the outcome of shoulder arthroplasty exist on this particular patient group.

**Case presentation:**

We included four dysmelic patients (five shoulders) with substantial glenoid dysplasia in a prospective database after approval by the local ethics committee. Once conservative treatment options had been exhausted, the patients were treated with shoulder arthroplasty and assessed clinically and radiographically before and after surgery. The mean patient age at the time of surgery was 50.4 years. The minimum follow-up time was 24 months (24–91 months). All patients experienced a considerable improvement of range of motion (ROM) and a relief of pain. No intra- or postoperative complications appeared.

**Conclusion:**

Patients with dysmelia have acceptable short and mid-term results with resurfacing hemiarthroplasty. It is an effective although somewhat complicated method to relieve pain and improve movement. Long-term performance of arthroplasty in patients with dysmelia remains to be seen, particularly with regard to the remaining problem of the altered and often deficient glenoid.

## Background

The cases of people with thalidomide induced phocomelia serve as an example as 2012 was the 50th anniversary of one of the most tragic occurrences in the history of modern medicine. Today the victims of thalidomide have grown up and suffer from severe physical problems. Their average age now is between 52 and 57 years. Gradually, restriction of mobility and increasing pain result in a diminished quality of life of the affected young people [1, 2]. The combination of several factors promote the development of osteoarthritis, as life expectancy has increased in this population. Anatomical anomaly of the upper limbs affects the bony anatomy of the proximal humerus as well as the glenoid as defined by dysplasia and the glenohumeral articular surface. Furthermore, it affects the soft tissue by means of the glenohumeral ligaments and capsule, the muscles themselves and their insertions. Overloading and overstressing lead to severe premature failure of the affected joints. Patients suffer from a reduced functional ROM, limb contractures and increasing pain, which lead to limited mobility and a loss of function in activities of daily routine. Patients develop alternative movements in order to remain independent of other people’s help and carry on tasks of daily living. Limited mobility of adjacent joints leads to extreme stresses on the shoulder joint, which attempts to compensate this disability. Despite intensive physical therapy, long-term sequelae have increased in our patients in recent years.

The primary aim of this study was to evaluate our experience with cases of glenohumeral osteoarthritis caused by dysmelia and treated with arthroplasty. Only a few articles report on the development of osteoarthritis in patients with glenoid dysplasia [3–5]. Our patient series showed similar signs of physical impairment and psychological distress. When the patients initially visited our clinic, the primary symptoms were increasing shoulder pain and a restricted ROM. Despite exploiting all conservative therapy options, joint function deteriorated, and pain increased over several years, leading to severe impairment necessitating invasive therapeutic measures.

### Thalidomide-induced phocomelia

Some 10.000–12.000 children in more than 46 countries all over the world were estimated to have been born with severe physical disabilities until 1962, a result of the sleep-inducing drug thalidomide, if it had been taken in the first trimester of pregnancy [6–8]. Thalidomide-induced embryopathy results in malformation of various degrees. The exact teratogenic mechanism of thalidomide causing limb malformations remains unclear [9, 10]. Children had abnormally short limbs, to some extent they showed an absence of the proximal portion of limbs and the distal parts being attached to the trunk - a condition which is known as phocomelia, which is more common in the upper extremities.

Literature supports our clinical impression that there is a growing need for information as the thalidomide victims are getting older and their problems in everyday life increase every day [2, 7]. The Department of Gerontology at the University of Heidelberg has recently evaluated 900 questionnaires of 2380 contacted thalidomide victims. It becomes apparent that one third of the respondents are already afflicted with recently diagnosed acquired osteoarthrosis of the shoulder, 58 % are afflicted with shoulder aches and about 40 % are suffering from a myasthenia or muscle hardening of the upper limbs. There is a significant increase of all disorders compared to results obtained 5 years ago [11].

## Case presentation

### Patients

This study investigates a consecutive series of four dysmelia patients who underwent shoulder arthroplasty at the University of Heidelberg. All clinical and radiographic data from these patients were evaluated retrospectively.

Three patients suffered from thalidomide-induced phocomelia. In one patient dysmelia occurred without a known cause. All patients suffered from increasing shoulder pain and a restricted ROM due to osteoarthritis and underwent arthroplasty in our hospital. All conservative therapy options had been exhausted. An arthrodesis was not considered an acceptable solution. Regular development of the shoulder and upper limbs was never observed. Additionally three patients showed pronounced ankylosis at the elbow and wrist joints. Excessive wear of the glenohumeral joint was observed in all patients. The wear process led to significant pain and impaired mobility, which even led to incapacity to work and provide for oneself and a diminished quality of life. Limitations extended over several years and the usual conservative measures were taken to delay the progression of the disease. Patients reported having had a long period of suffering. Humeral head resurfacing prostheses were used in all cases but one, in which a stemless prosthesis was used. All patients agreed with the publication of their clinical course (Ethics committee vote: S-305/2007, Ethikkommission Medizinische Fakultät Heidelberg). The subjects were assessed clinically before and after surgery quantifying their ROM and the pain on the visual analog scale (VAS). ROM was quantified with a goniometer.

### Preoperative diagnostics

**Fig. 1:**
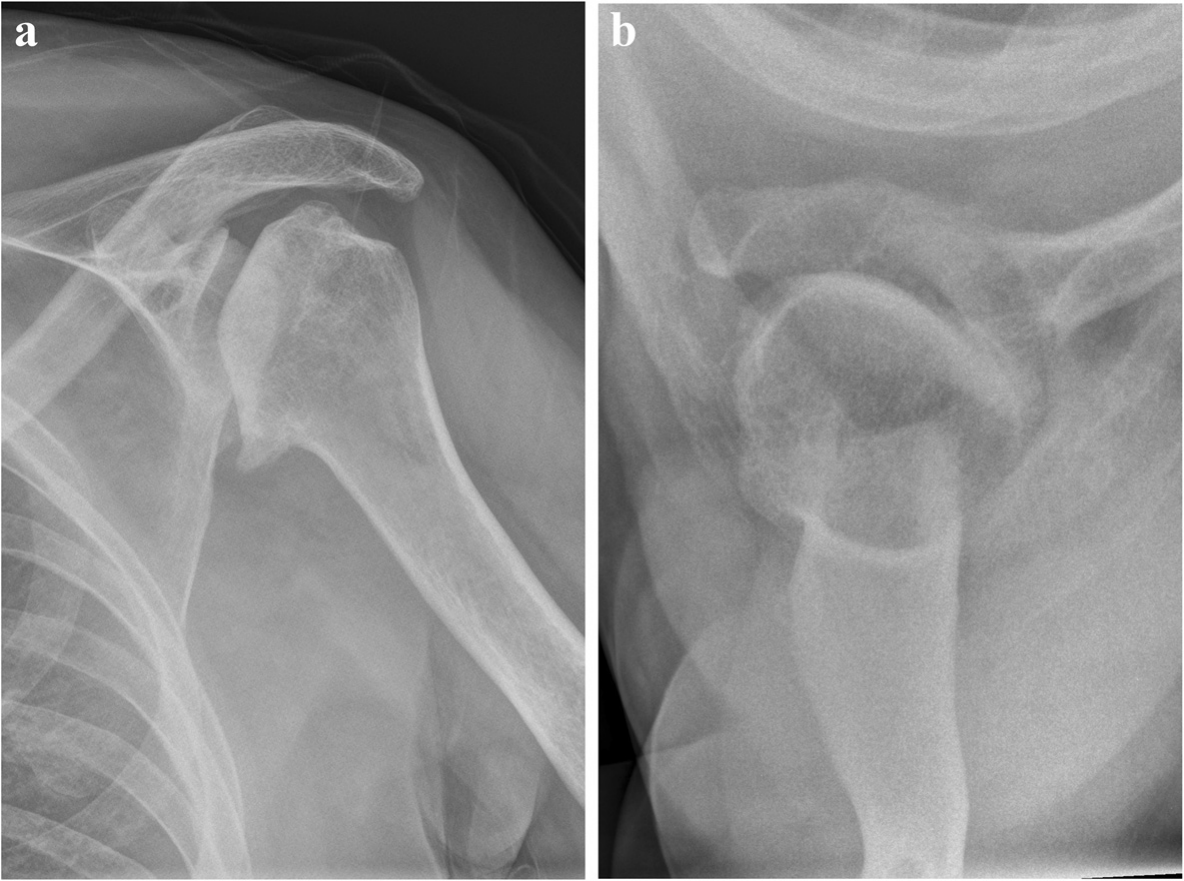
**a** and **b**: Preoperative true AP and axial view of the left shoulder show the displacement of the joint and hypoplasia of the glenoid. X-rays reveal a small, irregularly shaped bone (Case 3). Poor ROM prevents optimal visualization of the bone stock and the glenoid surface

Standard radiological diagnostics include conventional x-ray images: true anterior-posterior (AP) view (Fig. 1a) and axial view (Fig. 1b).

**Fig. 2:**
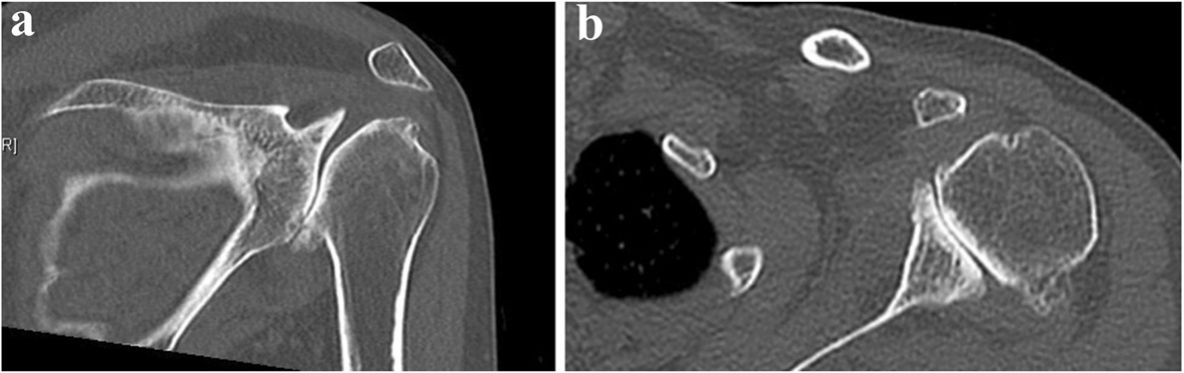
**a** and **b**: The cross-sectional pictures of the shoulder demonstrate the preoperative wear of all parts of the glenohumeral joint with a hypoplastic glenoid (Case 3). A large inferior socket defect can be seen. (**a** - coronal plane. **b** - axial plane)

The dysplastic glenoid cavity appeared irregularly shaped. The humeral heads were deformed and flattened in this region. An assessment of regional anatomy was limited by severe defects of the shoulder joint and necessitated performing computed tomography (CT) (Fig. 2a and b).

### Surgical technique and implants

The operation was performed in beach chair position under general anaesthesia through the standard anterior deltopectoral approach using the technique described by Neer [12]. If the long biceps tendon was present, a tenotomy in addition to extensive capsular tissue release was performed. Of note, none of these patients had a well-defined rotator cuff, but all had a substantial joint capsule constriction and adhesion to surrounding structures. Peripheral osteophytes were removed from the humeral head in order to enable the necessary dislocation. The status of the rotator cuff, the humeral head and the glenoid cavity were used to determine the final implant. Medialization of the glenohumeral joint and an Outerbridge chondromalacia grade III to IV was noted in all patients [13]. Only one patient showed an intact glenoid socket suitable for attachment of a glenoid component (Aequalis Glenoid medium, Tornier Inc., Edina, MN, USA, Case 4, Fig. 5). A correct central placement was targeted. Reaming was performed without opening the cancellous bone. An individualized glenoid component was not necessary. All other patients received hemi-arthroplasty due to a combination of substantial bone stock defect and dysplastic development and misalignment of the glenoid, making reconstruction impossible.

**Fig. 3:**
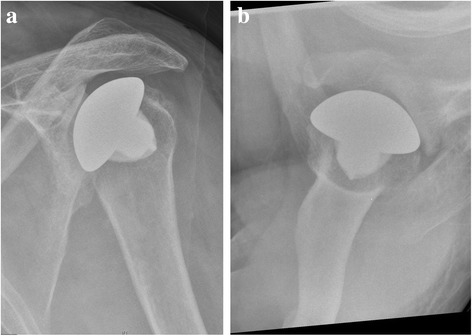
**a** and **b**: Postoperative pictures as an example for a patient (Case 3) without glenoid component of due to substantial bone stock defect (true AP and axial view of the left shoulder)

Cup prostheses were used in all cases but one, in which an anatomic prosthesis (Simpliciti®, Tornier Inc., Edina, MN, USA) was used (Case 3, Fig. 3). Aequalis® Resurfacing Head (Tornier Inc., Edina, MN, USA) was used twice (Cases 1 and 2). One case was treated with an Epoca RH Cup (Argomedical®, Cham, Switzerland) and a glenoid component (Aequalis®, Tornier Inc., Edina, MN, USA; Case 4). When using the resurfacing prosthesis, the humeral head was measured and a central guide pin was placed through the favoured axis before preparing the head with hemispherical reamers. The cement-free prostheses were impacted in press-fit technique in a slight valgus position. Before implanting the anatomic prosthesis an anatomical neck resection was performed. The centre of the head was located and a low profile collar was inserted before the definitive head was implanted. After implantation, the anterior capsule and the presumed subscapularis tendon were repaired using non-absorbable sutures.

### Postoperative rehabilitation

After surgery the arm was immediately placed in a splint (Tricodur® Gilchrist plus, BSN medical, Hamburg, Germany) for 2 weeks and all patients underwent a standard protocol for physical therapy with a rehabilitation programme. Active and passive exercises were limited to 90° as to abduction and flexion. External rotation was prohibited postoperatively for 4 weeks. After that time, free active and passive movement was allowed without any limitation.

### Statistical analysis

The statistical analysis was done with SPSS 20.0 software (IBM SPSS Statistics, Munich, Germany).

Usually a non-parametric test like the Wilcoxon rank sum test should be used for a small number of cases. From other studies it is well known that these parameters are normally distributed in the population, so we decided to use the t-test as it has better discriminatory power. The paired t test was used to compare the preoperative and postoperative scores. Mean values and the standard deviations (SD) were calculated. Values of *p* < 0.05 were considered significant.

## Results

### Clinical outcome

The findings of the preoperative and postoperative clinical examinations are shown in the following graphs and Tables 1, 2 and 3.

**Table 1:** Individual preoperative findings

Patient [No./side]	Age at time of arthroplasty [years]	ROM				VAS
		Flexion [°]	Abduction [°]	External rotation [°]	Internal rotation [region]^a^	
1 right	47	30	30	0	gluteal muscle	8/10
1 left	48	90	90	30	sacrum	7/10
2 left	50	40	60	−10	lateral thigh	6/10
3 left	51	20	35	−45	lateral thigh	8/10
4 left	58	20	90	10	lateral thigh	8/10

^a^Internal rotation was graded according to the posterior spinal region that could be reached by the thumb

**Table 2:** Individual postoperative findings

Patient [No./side]	Follow-up [months]	ROM				VAS
		Flexion [°]	Abduction [°]	External rotation [°]	Internal rotation [region]^a^	
1 right	60	140	120	55	L2	0/10
1 left	49	140	120	45	L3	0/10
2 left	32	160	130	10	L5	0/10
3 left	24	110	90	15	gluteal muscle	3/10
4 left	91	170	170	25	gluteal muscle	1/10

^a^Internal rotation was graded according to the posterior spinal region that could be reached by the thumb

**Table 3:** Preoperative and postoperative findings

	Preoperative^a^	Postoperative^a^	*p* value**
Constant score (points)	11.2 ± 5.3 (7 to 20)	78.4 ± 13,8 (54 to 88)	.0006
VAS (points)	7.4 ± 0.9 (6 to 8)	0.8 ± 1.3 (0 to 3)	.0002
Flexion (deg)	40.0 ± 29.2 (20 to 90)	144.0 ± 23.0 (110 to 170)	.0033
Abduction (deg)	61.0 ± 28.8 (30 to 90)	126.0 ± 28.8 (90 to 170)	.0034
External rotation (deg)	−3.0 ± 27.7 (−45 to 30)	30.0 ± 19.4 (10 to 55)	.0306
Internal rotation (deg)	−53.0 ± 17.2 (−80 to −40)	−76.0 ± 8.9 (−80 to −60)	.0402

**Preoperative compared with postoperative. The level of significance was set at *p* < 0.05

^a^The values are given as the mean and the standard deviation, with the range in parentheses

**Fig. 4:**
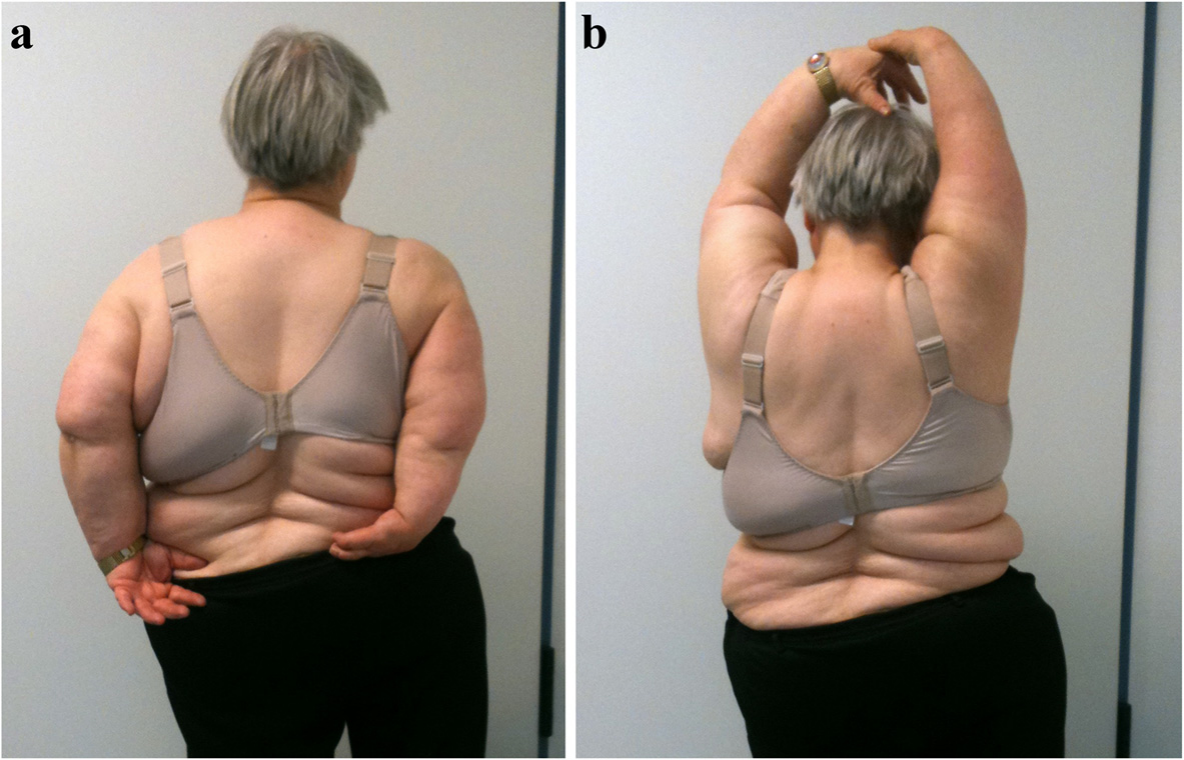
**a** and **b**: Clinical examination shows excellent ROM despite ankylosis of the elbow at follow-up (Case 4 - left shoulder)

All patients benefited from almost complete remission of pain and significant improvement of ROM. Only Cases 3 and 4 reported occasional pain of the shoulder, particularly in terminal motions. None of the patients required reoperation. No other intraoperative or postoperative complications like wound infection, hematoma or neurological deficiencies occurred (Fig. 4).

Furthermore, the Constant score was calculated [8]. The mean Constant score improved from 11.2 points (7 to 20 points) preoperatively to 78.4 points (54 to 88 points) postoperatively. Flexion and abduction were significantly higher. Significant differences were also found in external and internal rotation.

### Radiological outcome

**Fig. 5:**
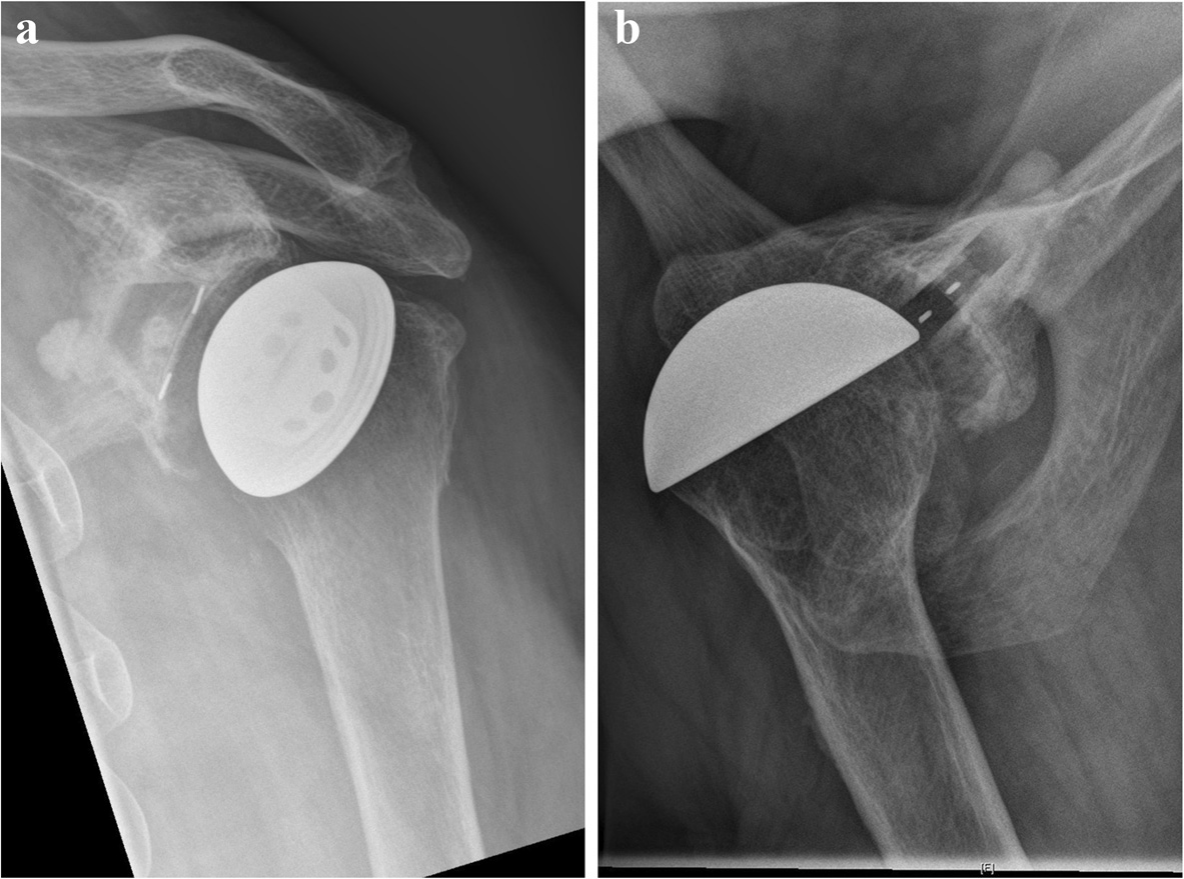
**a** and **b** show total shoulder arthroplasty (Case 4) at follow-up (true AP and axial view of the left shoulder)

Radiographical signs of implant loosening (radiolucent lines around the humeral implants or osteolysis) were not found in our group. Neither radiolucent lines nor signs of loosening were found around the glenoid component in Case 4. Neither malposition nor humeral head dislocation was noted. In our thalidomide patients, the humeral head was concentrically reduced in the deficient glenoid. No signs of emerging glenoid erosion or wear were found in postoperative x-rays. No heterotopic ossification was found. No intra- or postoperative complications appeared. None of the patients required reoperation (Fig. 5a and b)

.

## Discussion

### Arthroplasty for patients with dysmelia

Patients with glenohumeral joint dysplasia have limited treatment options. Smaller, altered anatomy and fibrosis of the capsule and ligaments make surgery extraordinarily difficult. In addition, the treatment must adapt to the requirements of life and delay the progression of the disease. Arthrodesis of the shoulder would reduce pain. However, in combination with coexistent ankylosis of the elbow or wrist joint, it results in a total loss of functionality of the limb. Arthroplasty with stemmed or reverse shoulder prosthesis is not an adequate procedure for patients with glenohumeral joint dysplasia. New concepts for anatomical reconstruction have been developed over the years to restore anatomy without loss of the humeral bone stock [14–16]. Humeral resurfacing arthroplasty can be an alternative to conventional arthroplasty. Resurfacing arthroplasty entails less invasive surgery, shorter operation times, little to no risk of periprosthetic stem fractures or loosening of the glenoid component and preservation of bone stock without loss of humerus length. This allows for easier revision surgery, if necessary. Meanwhile, humeral head resurfacing prostheses have been used in many shoulder diseases including primary and posttraumatic osteoarthritis, avascular necrosis, rheumatoid arthritis and cuff-tear arthropathy. These indications have proven useful in younger patients [17, 18], but are not suitable for advanced necrosis or deformation of the humeral head [19]. Due to anatomic anomalies - most notably dysplasia of the glenoid and no well-defined rotator cuff - a total shoulder prosthesis could not be implanted. Anchoring a glenoid component, either anatomical or reverse, was not feasible due to the lack of glenoid bone stock. In addition, it was not possible to use bone-sparing glenoid components as suggested by Sears et al. or to reconstruct the socket of the glenoid in all three thalidomide patients [20]. Consequently, the patients received hemiarthroplasties. In order to expose the anatomic neck of the proximal humerus and to excavate humeral head osteophytes, an improvement of external rotation using a sufficient soft tissue release was performed. The decisive argument for resurfacing arthroplasty is the possibility of having the free choice of retroversion as well as inclination angle. The lack of surgical landmarks requires substantial experience to avoid imprecise positioning of the prosthesis and improper soft tissue balancing. The difficulty in achieving arthroplasty stability is to perform a balancing act in the management of the bone and soft tissue in dysmelic patients.

Arthroplasty among patients with glenohumeral dysmelia is a rarely performed surgery, and as a result no long-term results are available. Only one source, Newman et al., reported a case of a 35-year-old woman with thalidomide-induced phocomelia. In 1999 he had predicted a rise of degenerative joint diseases in this population [21]. Duralde et al. reported on two cases with Apert’s Syndrome and Erb’s palsy treated with resurfacing prostheses [22]. ROM did not change significantly at follow-up, but arthroplasty resulted in high patient satisfaction and excellent pain relief. Mansat et al. treated 4 patients with not otherwise specified dysmelia using cup prostheses without glenoid resurfacing, even in cases of eccentric glenoid wear. They reported, that the worst results were obtained for rheumatoid arthritis and dysplasia (Constant 60 points; follow-up 33.7 months; mean age 62 years). A pain score of 11.8 points was reported according to Constant criteria of 15 points [23]. This correlates with our patient series, which reported a mean pain of 13 points according to Constant criteria. Smith et al. reported on three cases of hemiarthroplasty with stemmed prostheses in a series of twelve patients with primary glenoid dysplasia [24]. The mean age at onset of symptoms was 50 years with mean pain ratings on the visual analogue score of 5 out of 10 postoperatively. The follow-up time was not described. Due to of the lack of glenoid bone stock, inserting a glenoid component would have been difficult and the results were described as relatively disappointing compared with standard procedures. The premature development of osteoarthritis in patients with primary dysplasia of the glenoid has been described in the literature [25, 26]. Allen et al. reported an update to the series by Sperling et al. [3]. They treated 22 shoulders with glenoid dysplasia and secondary osteoarthritis using eight hemiarthroplasties. The authors recommended that glenoid deficiency and cartilage wear should be addressed at the time of shoulder arthroplasty in patients with glenoid dysplasia and glenoid problems necessitating revision surgery were frequent. Our experience would confirm this. We performed glenoid replacement in only one case in which a bone stock of the glenoid was suited to accept a glenoid component. Both Smith et al. and Allen et al. reported on a specific disease limited to the glenoid, in contrast to our series where the surrounding soft tissue was also involved [24, 27].

Sewell et al. [5] described a series of 13 shoulders in 10 patients. Five unconstrained TSAs, 4 linked (constrained) TSAs, and 4 HAs (2 stemmed and 2 resurfacing implants) were implanted. He was able to prove that arthroplasty provides the ability to improve pain and function and concluded that shoulder arthroplasty is a viable treatment option in patients with skeletal dysplasia. He explained that their high revision rate of 31 % was due to multiple pathologic joint processes compared to the general population.

A limitation of this study is the low incidence of disease, resulting in the small sample size, and making a randomized controlled setup unfeasible. The particular and varied shoulder anatomy of the individual patients is the reason for the heterogeneity of the prosthetic selection, which we feel is necessary to offer the patient the best treatment.

## Conclusion

This patient series indicates that shoulder arthroplasty is a feasible treatment option for patients with dysmelia who develop osteoarthritis of the shoulder. Short to midterm results support the procedure of humeral head resurfacing. Resurfacing and stemless shoulder arthroplasty seem to be a promising option to treat non-anatomical conditions, especially if conservative measures have been exhausted and arthrodesis is not desired. A considerable improvement of ROM and pain reduction was achieved in all patients. Prior to surgery the expectations of an operation and personal demand must be clarified because arthroplasty cannot become a substitute for emerging impaired mobility, improper use or mechanical overstressing. However, long-term performance of arthroplasty in patients with dysmelia remains to be seen.

### Consent

Written informed consent was obtained from all the patients for publication of this case report and any accompanying images. A copy of the written consent is available for review by the Editor of this journal. All patients agreed with the publication of their clinical course (Ethics committee vote: S-305/2007).

## Abbreviations

AP: anteroposterior
CT: computed tomography
ROM: range of motion
VAS: visual analog scale
